# Factor Xa inhibitors versus warfarin in patients with non-valvular atrial fibrillation and diabetes mellitus: a systematic review and meta-analysis of randomized controlled trials

**DOI:** 10.1097/MS9.0000000000001621

**Published:** 2024-01-03

**Authors:** Mohammad M. Zahoor, Saad Mazhar, Aima Azhar, Fasih Mand Khan, Usama Anees, Rimsha R. Vohra, Umer Ejaz, Sayed Jawad

**Affiliations:** a Department of Medicine, Lahore Medical and Dental College; b Department of Medicine, King Edward Medical University; c Department of Medicine, Fatima Jinnah Medical University; d Department of Surgery, Fatima Memorial College of Medicine and Dentistry, Lahore; e Department of Medicine, Quad-e-Azam Medical College, Bahawalpur; f Department of Medicine, Dow University of Health Sciences, Karachi; g Department of Medicine, Rawalpindi Medical College, Rawalpindi, Pakistan; h Department of Medicine, Kabul University of Medical Sciences, Kabul, Afghanistan

**Keywords:** factor Xa, non-valvular atrial fibrillation, warfarin

## Abstract

**Background:**

Patients with non-valvular atrial fibrillation with diabetes face increased stroke and cardiovascular risks. This study compares factor Xa inhibitors and warfarin using data from randomized controlled trials (RCTs).

**Methods:**

MEDLINE, Embase, and Cochrane CENTRAL databases were searched for RCTs comparing the risk of efficacy and safety of any factor Xa inhibitors with dose-adjusted warfarin by diabetes status. Incidence of stroke/systemic embolism, major bleeding, intracranial hemorrhage, ischemic stroke, all-cause mortality, risk of hemorrhagic stroke, and myocardial infarction were among the outcomes of interest. A generic inverse-weighted random-effects model was used to calculate hazard ratios (HRs) with 95 percent confidence intervals (CIs).

**Results:**

After applying exclusion criteria, four RCTs containing 19 818 patients were included in the analysis. Compared with warfarin, meta-analysis showed statistically significant reduction in incidence of stroke/systemic embolism (HR 0.80 [95% CI 0.69–0.92]; *P*=0.002), intracranial hemorrhage (HR 0.49 [95% CI 0.37–0.65]; *P*<0.001), and risk of hemorrhagic stroke (HR 0.37 [95% CI 0.20–0.66]; *P*=0.001) in patients on factor Xa inhibitors. However, there was no discernible difference between two treatment arms in incidence of major bleeding (HR 0.93 [95% CI 0.84–1.04]; *P*=0.19), ischemic stroke (risk ratio (RR) 0.90 [95% CI 0.73–1.12; *P*=0.34), myocardial infarction (RR 0.88 [95% CI 0.67–1.15]; *P*=0.35), and all-cause mortality (RR 0.89 [95% CI 0.79–1.01]; *P*=0.06).

**Conclusion:**

Factor Xa inhibitors show a favorable balance between efficacy and safety compared with warfarin, which is consistent across a wide range of patients with atrial fibrillation known to be at high risk for both ischemic and bleeding events.

## Introduction

Highlights

- Factor Xa inhibitors reduce the risk of stroke/systemic embolism.
- Factor Xa inhibitors decrease intracranial hemorrhage, minimizing bleeding complications.
- Factor Xa inhibitors are effective in a population susceptible to ischemic and bleeding events.

In addition to an elevated risk of atrial fibrillation (AF), diabetes is associated with a worsening of symptoms, mortality, hospitalization, and quality of life^1^. Thromboembolic and hemorrhagic events were significantly elevated in AF patients with diabetes, indicating that diabetes should be considered a significant determinant in the CHA_2_DS_2_-VASc scoring systems for bleeding risk that are routinely applied to AF patients^2^. A prior meta-analysis of the four novel oral anticoagulant (NOAC) trials for stroke/systemic embolism (SSE) or severe bleeding found no significant interaction between therapy and diabetes status^3^. In diabetic patients with non-valvular atrial fibrillation (NVAF), current global recommendations recommend the use of NOACs as safer, more convenient, and effective alternatives to warfarin^4^.

Non-vitamin K antagonist oral anticoagulants (NOACs) represent a notable progression in antithrombotic approaches targeted at averting thromboembolic complications (e.g. SSE) in patients diagnosed with NVAF. These more recent agents are characterized by their consistent dosing and response, ability to regulate clot formation, fixed dosages, elimination of the requirement for monitoring, minimal drug interactions, absence of food interactions, and predictable onset and offset of action. NOACs were associated with a 14% relative reduction in severe bleeding and a 19% relative decrease in the composite endpoint, which included any SSE, in randomized phase III trials comparing them to warfarin treatment^5^. The clinical significance of the inquiry into whether non-steroidal anticoagulants (NOACs) maintain their effectiveness in preventing thromboembolic complications and their superior safety profile in comparison to warfarin is underscored by the propensity for both thromboembolic and hemorrhagic events associated with diabetes^4^. Regarding the primary outcome measures, phase III trials did not observe any interaction between diabetes status and pharmacological approach (NOACs versus warfarin). Nevertheless, the power of those individual studies may have been insufficient to assess the clinical efficacy of NOACs in specific patient subgroups, especially for endpoints with low incidence. To shed light on this matter, pooled analyses of data from multiple investigations may be beneficial^6^. In order to add more robust evidence regarding the differential degree of effectiveness and safety of these newer agents in patients with DM versus those without DM, particularly for rare adverse events (i.e. intracranial bleeding, ischemic stroke, or vascular death), we performed a meta-analysis of randomized trials comparing NOACs and warfarin in patients with NVAF. Consequently, we determined the absolute risk reduction of the outcome measure.

Regarding therapeutic efficacy, empirical research indicates that NOACs exhibit superior performance compared to warfarin^7^. Nonetheless, information regarding the danger of major bleeding has been notably inconsistent^8^. In contrast, some research has concluded that NOACs do not pose a significant risk of severe bleeding when compared to warfarin^7,9–11^. Based on the results of subgroup analyses of randomized controlled trials (RCTs), we conducted this systematic review and meta-analysis to investigate further the efficacy and safety of NOACs in the treatment of patients with NVAF and diabetes.

## Methods

This meta-analysis was conducted in accordance with the Preferred Reporting Items for Systematic Review and Meta-Analyses (PRISMA, Supplemental Digital Content 1, http://links.lww.com/MS9/A336) and Risk of Bias in Systematic Reviews and Assessment of Multiple Systematic Reviews (AMSTAR, Supplemental Digital Content 2, http://links.lww.com/MS9/A337) 2 guidelines^12,13^. The National Institute for Health Research (NIHR) is responsible for maintaining the International Prospective Register of Systematic Reviews (PROSPERO). This study was registered in NIHR PROSPERO (ID: CRD42023432132). In light of the fact that the information was publicly available, approval from an institutional review board (IRB) was unnecessary.

### Data sources and search strategy

Two independent reviewers conducted a comprehensive search of MEDLINE, Embase, and Cochrane CENTRAL from their inception until July 2021 (A.Z. and S.M.). Studies were extracted using abstracts and titles as criteria. A complete text evaluation was requested when necessary. MeSH (Medical Subject Headings) phrases and keywords were employed to identify brand names and generic forms of anticoagulant drugs, as well as symptoms associated with NVAF and diabetes. The comprehensive search strategy is provided in Supplementary Table 1 (Supplemental Digital Content 3, http://links.lww.com/MS9/A338).

### Study selection

The following types of studies were considered for inclusion: (1) RCTs that compared the safety and efficacy of non-steroidal anti-oxidant (NOAC) medications with dose-adjusted warfarin in different interventional arms, with or without diabetes status; (2) subgroup analyses of RCTs that examined the safety and efficacy of NOACs with dose-adjusted warfarin in relation to diabetes status; and (3) investigations into NOACs such as apixaban, dabigatran, edoxaban, and rivaroxaban. The following were the criteria for exclusion: (1) studies lacking corresponding outcome indicators, (2) duplicate results originating from the same population, and (3) studies lacking pertinent data despite attempts to contact the original author. In order to eliminate any duplicates, all articles were subsequently imported into Endnote Reference Library (Version X7.5; Clarivate Analytics, Philadelphia, Pennsylvania) software.

### Data extraction and assessment of study quality

Two reviewers (A.Z. and S.M.) extracted data independently from the chosen studies, including study characteristics, patient demographics, incident summaries, event counts, sample sizes, and treatment types. In addition, summary events pertaining to the desired outcomes were extracted, and risk ratios (RRs) accompanied by 95% confidence intervals (CIs) were computed from them. Study design, year of publication, number of test and control groups, age of test subjects and dosage of test medications, CHADS_2_ score, participant baseline characteristics, and methods used to identify and verify the diagnosis of NVAF and diabetes were extracted as data. The Cochrane Risk of Bias Tool (CRBT) was utilized to assess the quality of studies according to the following six categories: selection bias, performance bias, detection bias, attrition bias, reporting bias, and other bias.

### Statistical analysis

For all statistical calculations, RevMan (version 5.3; Nordic Cochrane Centre, The Cochrane Collaboration, Copenhagen) was utilized. Hazard ratios (HRs) with 95% CIs were compiled using Mantel–Haenszel (MH) random effects weighted methods. The heterogeneity among investigations was evaluated through the utilization of Higgins *I*^2^. Egger’s regression test was employed to assess the potential for publication bias. In light of the limited number of studies included, funnel plots were not employed to assess publication bias.

## Results

### Literature search and characteristics of included studies

**Figure 1:**
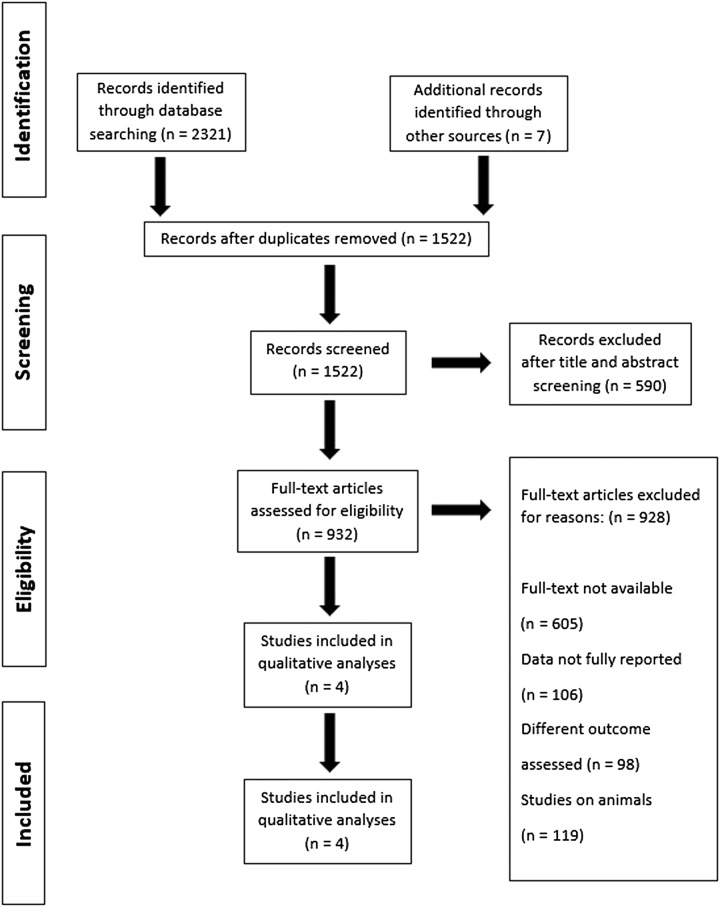
PRISMA (Preferred Reporting Items for Systematic Review and Meta-Analyses) flow diagram of study identification for meta-analysis.

The PRISMA flow diagrams (Fig. 1) illustrate the process of literature search and research selection. Four RCTs involving 19 818 patients were selected from the initial 2321 articles for this analysis, following the application of exclusion criteria. The demographic and baseline characteristics are detailed in Table 1. Regarding publication bias, Egger’s regression test yielded insignificant results (*t*=1.14; *P*=0.912).

**Table 1:** Baseline characteristics of included studies.

Author, year	Study design	Study site	Type of diabetes	Experimental group, NOACs	Control group, warfarin	Follow-up (years)
Bansilal *et al*., 2015^14^	Subgroup of RCT	International multicenter	Types 1 and 2	Rivaroxaban (*n*=2878, 16% received low dose)	2817	1.9
Brambatti *et al*., 2015^15^	Subgroup of RCT	International multicenter	Types 1 and 2	Dabigatran 110 mg twice daily (*n*=1409) Dabigatran 150 mg twice daily (*n*=1402)	1410	2
Ezekowitz *et al*., 2015^16^	Subgroup of RCT	International multicenter	Types 1 and 2	Apixaban (*n*=2559, NR percentage of low dose)	2263	1
Plitt *et al.*, 2020^17^	Subgroup of RCT	International multicenter	Types 1 and 2	Edoxaban (*n*=2559, NR percentage of low dose)	2521	NR

NOAC, novel oral anticoagulants; NR, not reported; RCT, randomized controlled trial.

### SSE

**Figure 2:**
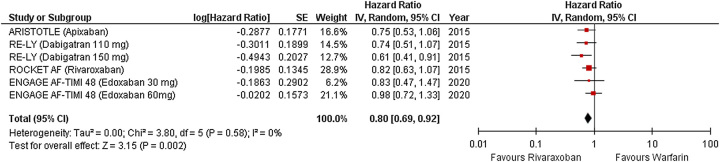
Forest plot showing results of factor Xa versus warfarin on stroke/systemic embolism (SSE).

Four studies reported the outcome of SSE (Fig. 2). Factor Xa inhibitors significantly reduced the risk of SSE compared with warfarin (HR 0.80 [95% CI 0.69–0.92]; *P*=0.002, *I*^2^=0%).

### Major bleeding

**Figure 3:**
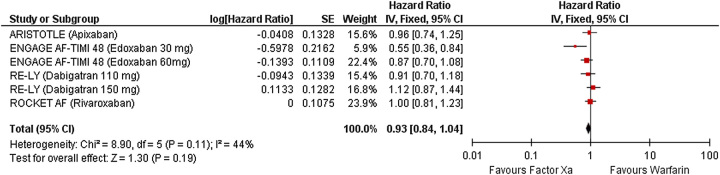
Forest plot showing results of factor Xa versus warfarin on major bleeding.

Four studies reported data on major bleeding (Fig. 3). Compared with patients on warfarin, patients on factor Xa inhibitors did not significantly alter the risk of major bleeding in patients with NVAF with diabetes. (HR 0.93 [95% CI 0.84–1.04]; *P*=0.19, *I*^2^=44%).

### Ischemic stroke

**Figure 4:**
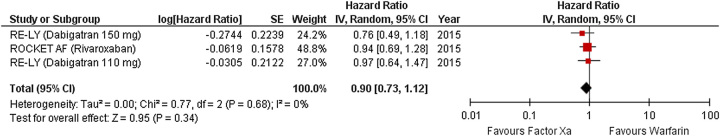
Forest plot showing results of factor Xa versus warfarin on ischemic stroke.

Three studies reported events on ischemic stroke. In terms of preventing ischemic stroke, there was no discernible difference between factor Xa inhibitors and warfarin (RR 0.90 [95% CI 0.73–1.12; *P*=0.34, *I*^2^=0%) (Fig. 4).

### Intracranial hemorrhage

**Figure 5:**
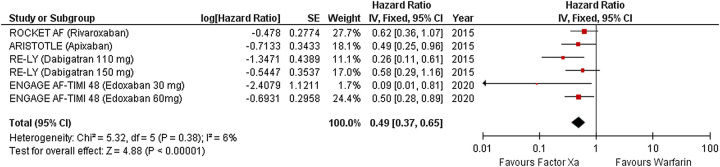
Forest plot showing results of factor Xa versus warfarin on hemorrhagic stroke.

Four studies reported intracranial hemorrhage (Fig. 5). Compared with warfarin, factor Xa significantly reduced the risk of intracranial hemorrhage (RR 0.49 [95% CI 0.37–0.65]; *P*<0.001, *I*^2^=6%).

### All-cause mortality

**Figure 6:**
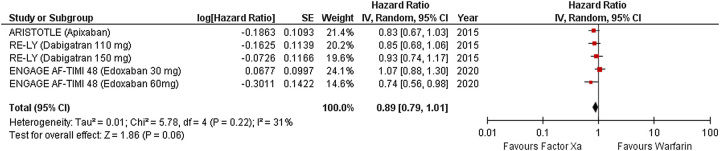
Forest plot showing results of factor Xa versus warfarin on intracranial bleeding.

Three RCTs provided data on all-cause mortality (Fig. 6). No discernible difference in risk of all-cause mortality was found between individuals on factor Xa inhibitors and warfarin, according to a meta-analysis (RR 0.89 [95% CI 0.79–1.01]; *P*=0.06, *I*^2^=31%).

### Myocardial infarction

**Figure 7:**
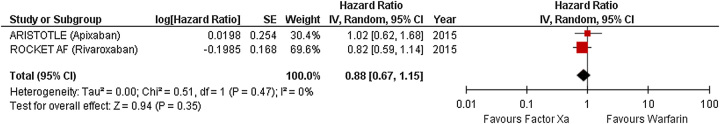
Forest plot showing results of factor Xa versus warfarin on gastrointestinal bleeding.

Two studies provided data on myocardial infarction (Fig. 7). No discernible difference in risk of myocardial infarction was found between individuals on factor Xa inhibitors and warfarin, according to a meta-analysis (RR 0.88 [95% CI 0.67–1.15]; *P*=0.35, *I*^2^=0%).

### Risk of hemorrhagic stroke

**Figure 8:**
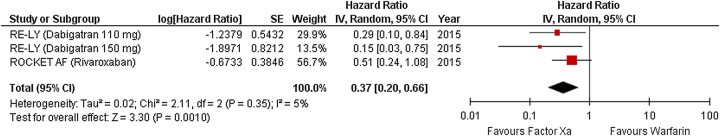
Forest plot showing results of factor Xa versus warfarin on vascular death.

Two studies provided data on the risk of hemorrhagic stroke (Fig. 8). Compared with warfarin, factor Xa inhibitors reduced the risk of hemorrhagic stroke in patients with NVAF with diabetes (HR 0.37 [95% CI 0.20–0.66]; *P*=0.001, *I*^2^=5%).

### Quality assessment

According to the Cochrane risk-of-bias methodology for randomized trials, RCTs were rated as having a moderate risk of bias (Supplemental Figure 1, Supplemental Digital Content 3, http://links.lww.com/MS9/A338).

## Discussion

In patients with NVAF, the prevalence of diabetes substantially elevates the risk of stroke by an estimated 70%^1^. Non-vitamin K antagonist oral anticoagulants (NOACs) have introduced several advantages over warfarin, including more predictable pharmacodynamics, fewer drug and food interactions, and the elimination of routine laboratory monitoring. Anticoagulation therapy is vital in preventing stroke in these patients^18^.

NOACs significantly reduce the incidence of stroke, including systemic embolism, ischemic stroke, hemorrhagic stroke, intracranial bleeding, gastrointestinal bleeding, myocardial infarction, and vascular death, in comparison to warfarin, according to this meta-analysis involving 267 272 patients. In contrast to warfarin, apixaban alone demonstrated a reduced likelihood of major bleeding; dabigatran, rivaroxaban, and edoxaban exhibited comparable hazards of major bleeding.

Further substantiating the superiority of NOACs over warfarin in mitigating the likelihood of systemic embolism was a comprehensive analysis of the RE-LY, ROCKET AF, ARISTOTLE, and ENGAGE AF-TIMI 48 trials^19^. In addition, previous research has shown that diabetic and nondiabetic patients with NVAF who take NOACs or vitamin K antagonists face comparable risks of severe bleeding and systemic embolism^19^. Consistent with these results, the present study indicates that NOACs reduce the risk of systemic embolism in patients with NVAF and diabetes without increasing the incidence of severe bleeding.

For the prevention of stroke in patients with NVAF, the 2018 European Heart Rhythm Association Room Fibrillation anticoagulation guidelines recommend NOACs as the treatment of choice^20^. As indicated by prior research, patients with NVAF and diabetes may find it more difficult and less compliant to adhere to anticoagulation standards while taking warfarin; therefore, NOACs are a preferable option for this population^19,21,22^.

Ruff *et al*.^23^ demonstrated through a meta-analysis that the administration of NOACs to patients with NVAF substantially decreased all-cause mortality. Nevertheless, an elevated incidence of gastrointestinal hemorrhage was also documented in their research regarding NOACs. The present study, on the other hand, found no difference between NOACs and warfarin in terms of all-cause mortality or gastrointestinal hemorrhage. The observed discrepancy could potentially be explained by the more rigorous inclusion standards applied in prior RCTs and the improved representation of the real-world population in the present investigation. NOACs did not offer a comparative advantage over warfarin in the prevention of ischemic stroke and intracranial hemorrhage, according to a study by Patti *et al*.^24^ These results contradict those of the present investigation. Potentially attributable to the larger sample size of the current study in comparison to the smaller sample size of the preceding study, variations may exist.

Critics of meta-analyses in this domain contend that although extensive phase 3 trials centered on the prevention of stroke in patients with AF possessed adequate power to evaluate the primary therapeutic effect of each individual drug in comparison to warfarin, they frequently lacked the statistical power to distinguish secondary outcomes and subgroups^25^. For example, in the analysis of all-cause mortality, it was found that only apixaban and low-dose edoxaban exhibited substantial decreases, whereas the HRs for all medications (and doses) remained relatively consistent. The results of the meta-analysis provide evidence in favor of the proposition that the new oral anticoagulants, when considered collectively, decrease all-cause mortality in the populations that participated in the clinical trials by around 10%.

In addition to offering reliable estimates of secondary outcomes, meta-analyses possess the capability to improve precision in the assessment of the comparative advantages of novel oral anticoagulants within subgroups that are clinically significant. It is critical to acknowledge that there is substantial variation in the risk of stroke and hemorrhage among distinct patient populations diagnosed with AF^26^. Specific demographic groups, including those aged 75 years or older, those with a prior history of stroke, and those with renal dysfunction, are at heightened risk for experiencing both ischemic events and hemorrhage^27^. Nevertheless, these individuals are frequently underrepresented and their participation in trials is inconsistent. As a result, the assurance provided by each individual trial regarding the overall balance between efficacy and safety in these high-risk populations is limited. For instance, discrepancies in the percentage of participants exhibiting a CHADS_2_ score ranging from 3 to 6 were predominantly attributable to the divergent enrollment of patients who had experienced a transient ischemic attack or stroke previously^19^. This exhaustive meta-analysis establishes for the first time that the relative safety and efficacy of novel oral anticoagulants are uniform among a wide spectrum of susceptible patient populations.

Adherence to medication regimens is a critical determinant in determining the effectiveness and safety of drugs^28^. While non-steroidal anticoagulants (NOACs) did demonstrate enhanced adherence in comparison to warfarin and offer specific benefits in clinical implementation, the findings of the present study indicated that medication compliance was more prevalent among the entire population. Consequently, medical personnel must continue to reinforce patient education, raise patients’ awareness regarding adherence, and ensure the safe and effective administration of drugs in clinical practice^29^.

Certain constraints should be acknowledged with regard to this research. Further limitations may arise from the relatively small sample size and patient selection, which could prevent the comprehensive representation of patient characteristics and clinical scenarios in the results. Furthermore, variations in follow-up duration, study designs, and patient populations may have been present in the included studies, potentially introducing heterogeneity. As a result, additional research is required to validate and build upon these results, including real-world studies and RCTs on a larger scale.

## Conclusion

In comparison to warfarin, factor Xa inhibitors exhibit a more advantageous equilibrium between safety and efficacy. This is evident in a diverse group of patients diagnosed with AF, who are recognized to have an elevated susceptibility to both ischemic and bleeding events.

## Ethical approval

Ethical approval for this was not necessary because all the data used in this study is publicly available in the trials referenced within the manuscript.

## Consent

This study type is a systematic review and meta-analysis of previously published publicly available studies. We included studies that took written informed consent from the participants of the study.

## Sources of funding

Not applicable.

## Author contribution

M.M.Z. and A.Z.: conceived the idea and designed the study; S.M.: extracted the data, analyzed it, and created the illustrations; S.J.: critically revised the manuscript. All authors drafted the manuscript.

## Conflicts of interest disclosure

There are no conflicts of interest.

## Research registration unique identifying number (UIN)

1. Name of the registry: National Institute for Health Research (NIHR) International Prospective Register of Systematic Reviews (PROSPERO).
2. Unique identifying number or registration ID: CRD42023432132.
3. Hyperlink to your specific registration (must be publicly accessible and will be checked): crd.york.ac.uk/PROSPERO/display_record.php?RecordID=432132

## Guarantor

Sayed Jawad (Department of Medicine, Kabul University of Medical Sciences).

## Data availability statement

All the data used in this study is publicly available in the trials, which are referenced in the bibliography.

## Provenance and peer review

Not commissioned, externally peer-reviewed.

## Supplementary Material
