# Supplementary material for: Factor Xa inhibitors versus warfarin in patients with non-valvular atrial fibrillation and diabetes mellitus: a systematic review and meta-analysis of randomized controlled trials
Source: Ann Med Surg (Lond). 2024 Jan 3;86(2):986–93. doi: 10.1097/MS9.0000000000001621 (PMC10849443; doi:10.1097/MS9.0000000000001621)
Supplement: Supplementary file 3 [file ms9-86-0986-s003.docx]

**Supplementary Materials**

**Supplemental Figure 1.** Assessment of Risk of Bias of Included Studies


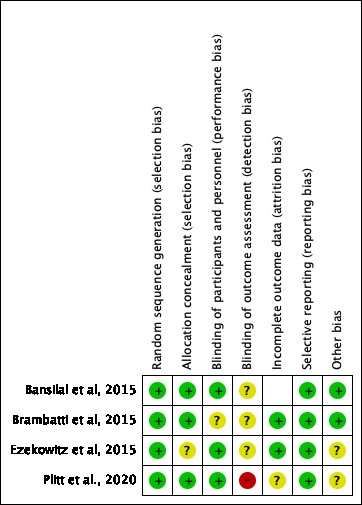


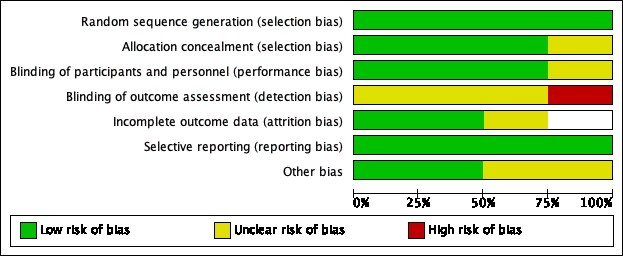


**Supplemental Table 1.** Search Strategy Used in Each Database

| **Database (Articles Retrieved)** | **Search Strategy** |
| --- | --- |
| MEDLINE  (146 results) | (("Factor Xa Inhibitors"[MeSH Terms] OR "Factor Xa Inhibitors"[All Fields]) AND ("warfarin"[MeSH Terms] OR "warfarin"[All Fields]) AND ("atrial fibrillation"[MeSH Terms] OR "atrial fibrillation"[All Fields]) AND ("diabetes mellitus"[MeSH Terms] OR "diabetes mellitus"[All Fields] OR "diabetes"[All Fields])) AND ("systematic review"[MeSH Terms] OR "systematic review"[All Fields]) AND ("meta-analysis"[MeSH Terms] OR "meta-analysis"[All Fields]) AND ("randomized controlled trials"[MeSH Terms] OR "randomized controlled trials"[All Fields]) |
| Embase  (56 results) | ("Factor Xa Inhibitors" OR "Factor Xa") AND ("warfarin") AND ("atrial fibrillation" OR "AF") AND ("diabetes mellitus" OR "diabetes") AND ("systematic review" OR "meta-analysis") AND ("randomized controlled trials" OR "RCT") |
| Cochrane Library  (25 results) | ("Factor Xa Inhibitors" OR "Factor Xa") AND ("warfarin") AND ("atrial fibrillation" OR "AF") AND ("diabetes mellitus" OR "diabetes") AND ("systematic review" OR "meta-analysis") AND ("randomized controlled trials" OR "RCT") |
